# The protective roles of allicin on type 1 diabetes mellitus through AMPK/mTOR mediated autophagy pathway

**DOI:** 10.3389/fphar.2023.1108730

**Published:** 2023-02-03

**Authors:** Rengcheng Qian, Huihui Chen, Hongzhou Lin, Yalan Jiang, Pingping He, Yinjuan Ding, Huilan Wu, Yongmiao Peng, Lingfei Wang, Congde Chen, Dexuan Wang, Weiping Ji, Xiaoling Guo, Xiaoou Shan

**Affiliations:** ^1^ Department of Pediatrics, The Second Schoozl of Medicine, The Second Affiliated Hospital and Yuying Children’s Hospital of Wenzhou Medical University, Wenzhou, Zhejiang, China; ^2^ Key Laboratory of Children Genitourinary Diseases of Wenzhou, The Second Affiliated Hospital and Yuying Children’s Hospital of Wenzhou Medical University, Wenzhou, Zhejiang, China; ^3^ Key Laboratory of Structural Malformations in Children of Zhejiang Province, The Second Affiliated Hospital and Yuying Children’s Hospital of Wenzhou Medical University, Wenzhou, Zhejiang, China; ^4^ Department of General Surgery, The Second Affiliated Hospital and Yuying Children’s Hospital of Wenzhou Medical University, Wenzhou, Zhejiang, China; ^5^ Basic Medical Research Center, The Second Affiliated Hospital and Yuying Children’s Hospital of Wenzhou Medical University, Wenzhou, Zhejiang, China

**Keywords:** type 1 diabetes mellitus (T1DM), allicin, pancreatic β cells, autophagy, AMPK/mTOR pathway

## Abstract

**Background:** Type 1 diabetes mellitus (T1DM) is one of the most common endocrine and metabolic diseases in children. Pancreatic β cells are thought to be critical cells involved in the progression of T1DM, and their injury would directly lead to impaired insulin secretion.

**Purpose:** To investigate the protective effects of allicin on pancreatic β cell injury and elucidate the underlying mechanism.

**Methods:** The streptozotocin (STZ)-induced mouse T1DM model *in vivo* and STZ-induced pancreatic β cell Min6 model in vitro were used to explore the effects of allicin on T1DM. The experiments include fasting blood glucose test, oral glucose tolerance detection, HE staining, immunohistochemistry, immunofluorescence, TUNEL staining, western blot, real-time quantitative PCR (RT-qPCR), and flow cytometry.

**Results:** Allicin could significantly decrease blood glucose level, improve islet structure and insulin expression, and inhibit apoptosis to reduce STZ-induced pancreatic β cell injury and loss through activating AMPK/mTOR mediated autophagy pathway.

**Conclusion:** Allicin treatment significantly reduced STZ-induced T1DM progression, suggesting that allicin may be a potential therapy option for T1DM patients.

## Introduction

Type 1 diabetes mellitus (T1DM) is characterized by insulin deficiency caused by autoimmune-mediated pancreatic β cell injury and loss. It is a chronic disease that causes hyperglycemia and is accompanied by polydipsia, polyphagia, polyuria, weight loss, headache, abdominal symptoms, ketoacidosis, and other symptoms (Group, 2004; Atkinson et al., 2014; Katsarou et al., 2017). According to the International Diabetes Federation, 8.8% of people worldwide have diabetes and 10%–15% of them are subjected to T1DM (Zinman, 2015). Long-term high blood glucose levels can cause microvascular complications including nephropathy, neuropathy, retinopathy, and subsequently cause a huge social and economic burden (Katsarou et al., 2017). At present, the clinical management of T1DM patients mainly focuses on intensive insulin therapy, with the purpose of keeping the blood glucose level as close to the normal as possible, improving the level of glycosylated hemoglobin and reducing complications (Nathan and Group, 2014). However, there are still certain limitations for insulin therapy. So it is extremely urgent to seek more effective drugs or approaches for T1DM treatment.

Autophagy, the type II form of programmed cell death, is thought to be an evolutionarily conserved process. The denatured or aged proteins and defective organelles are brought into lysosomes for degradation and play a crucial role in cell homeostasis (Lai et al., 2017). Previous studies have shown that the apoptosis of pancreatic β cells is an important part of the pathogenesis of T1DM (Kim et al., 1999), and autophagy may be involved in the regulation of apoptosis and the development of multiple stages of T1DM (Degenhardt et al., 2006; Vaquero et al., 2007; Wu et al., 2008). It is well-known that autophagy can be regulated by multiple signaling pathways and effector factors (Cheng et al., 2017). The accumulated evidence suggested that mTOR is a core regulator of autophagy (Zhai et al., 2018). It has been reported that cPKCγ deficiency can exacerbate autophagy damage and hyperphosphorylation accumulation of tau protein through AMPK/mTOR pathway, thereby improving neurocognitive impairment in T1DM (Zheng et al., 2022). T1DM management could be improved through regulating mTORC/TFEB/calcineurin axis pathway to promote lysosome production (Pan et al., 2020). Therefore, we speculated that AMPK/mTOR mediated autophagy pathway might be an important strategic target for T1DM treatment.

Garlic is one of the world’s common vegetables, and is used not only in cooking but also in traditional and modern medicine due to its medicinal properties, such as anti-diabetic, anti-cancer, anti-inflammatory, anti-bacterial, anti-oxidant, and anti-immunomodulatory activities (Capasso, 2013; Borlinghaus et al., 2014; Gao et al., 2019). The medicinal value of garlic for diabetes has attracted the attention of researchers. Allicin, as the main bioactive substance (Hayat et al., 2016), is produced by allinase conversion of allicine when garlic cloves are crushed. Allicin is absorbed efficiently, although part of it is metabolized as a rapidly absorbed intermediate, and Allyl methyl sulfide (AMS) is the main breath metabolite of allicin (Lawson and Hunsaker, 2018). Allicin is hydrophobic and can efficiently cross cell membranes without membrane leakage, fusion or aggregation (Nadeem et al., 2021). It enhances the effect of intracellular action (Miron et al., 2000). Recently, it has been suggested that allicin could reduce pathological cardiac hypertrophy through the autophagy pathway (Ba et al., 2019). Allicin has also been reported to attenuate oxidative stress induced by advanced oxidative protein products and mitochondrial apoptosis in human nucleus pulposus cells, thereby improving disc degeneration (Xiang et al., 2020). So far, there has been no report of allicin improving pancreatic β cells in T1DM patients. Therefore, we would try to investigate the protective effects of allicin on streptozotocin (STZ)-induced mouse T1DM model *in vivo* and STZ-induced mouse pancreatic β cell line (Min6) model *in vitro*, and illustrate the underlying mechanism.

## Materials and methods

### Reagents and instruments

Dulbecco’s modified Eagle’s medium (DMEM, 8122465, Shanghai, China), 1% penicillin/streptomycin (P/S, 15140122, Gibco, CA, United States), 10% fetal bovine serum (FBS, A3160801, Gibco, CA, United States), streptozotocin (STZ, S0130, Sigma-Aldrich, CA, United States), 0.1 M sodium citrate buffer (C1013, Solarbio, Beijing, China), glucometer (580, Yuwell, Beijing, China), mouse insulin enzyme linked immunosorbent assay (ELISA) kit (H203-1-1, Jiancheng, Nanjing, China), Hank’s Balanced Salt Solution (HBSS, H1025, Solarbio, Beijing, China), Ficoll gradient 1.077 (LTS1077, Solarbio, Beijing, China), Ficoll gradient 1.119 (LTS1119, Solarbio, Beijing, China), β-mercapto-ethanol (BY12286, Boyun, Shanghai, China), dimethylsulfoxide (DMSO, 733210, Sigma-Aldrich, CA, United States), compound C (CC, HY-13418A, MedChem Express Biotechnology, NJ, United States), Cell Counting Kit-8 (CCK-8, 40203ES80, Yeasen, Shanghai, China), microplate analyzer (Thermo, MA, United States), Hematoxylin-Eosin (HE) staining kit (G1120, Solarbio, Beijing, Chin), the peroxidase blocking agent (AR1108, Zsbio, Beijing, China), Goat anti-rabbit secondary antibody (S001, Affinity Biosciences, Melbourne, Australia), DAB color development kit (ZLI-9018, Zsbio, Beijing, China), Trizol (15596026, Invitrogen, Carlsbad, United States), ReverTra Ace qPCR RT Master Mix (FSQ-201, TOYOBO, Osaka, Japan), PrimeScript RT Master Mix (RR036A, TaKaRa, Kusatsu, Japan), RIPA lysis buffer (R0010, Solarbio, Beijing, China), phenylmethane-sulfonyl fluoride (PMSF) (P0100, Solarbio, Beijing, China), BCA Protein Assay Kit (P0010, Beyotime, Shanghai, China), polyvinylidene fluoride (PVDF, 88520, Thermo Fisher, MA, United States), skim milk (P0216, Beyotime, Shanghai, China), ECL color development kit (S0201, EpiZyme, Shanghai, China), ChemiDic™XRS imaging system (Bio-Rad Laboratories, CA, United States), 0.3% TritonX-100 (T9284, Sigma-Aldrich, CA, United States), Annexin V-PE apoptosis detection kit (556547, Becton Dickinson, NY, United States), EDTA-free trypsin solution (25200072, Gibco, CA, United States), Goat anti-rabbit secondary antibody (S002, Affinity Biosciences, Melbourne, Australia), 10% glucose (R00601, Leagene, Beijing, China), Allicin (HY-N0315, MedChem Express Biotechnology, NJ, United States), collagenase V (C9263, Sigma, CA, United States), Goat Serum (C0265, Beyotime, Shanghai, China), TdT mediated dUTP Nick End Labeling (TUNEL, 40308ES20, Yeasen, Shanghai, China), PAGE Gel Fast Preparation Kit (PG111, EpiZyme, Shanghai, China), PAGE Gel Fast Preparation kit (PG113, EpiZyme, Shanghai, China), FITC AffiniPure Goat Anti-Mouse IgG (H + L) (E031210-01, Earthox, LA, United States), FITC AffiniPure Goat Anti-Rabbit IgG (H + L) (E031220-01, Earthox, LA, United States), Cy3 AffiniPure Goat Anti-Rabbit IgG (H + L) (E031620-01, Earthox, LA, United States), Cy3 AffiniPure Goat Anti-Mouse IgG (H + L) (E031610-01, Earthox, LA, United States), Antifade Mounting Medium with DAPI (P0131, Beyotime, Shanghai, China), Flow cytometer (Beckman Coulter, CA, United States), and optical microscope (Nikon, Japan).

### Animal

6-week-old male BALB/c mice with 18–22 g were provided by Laboratory Animal Center, Wenzhou Medical University, Wenzhou, China. All mice were fed on a condition with 12 h light/dark cycle at 23°C ± 2°C and relative humidity of 45%–55%. All food and water were accessed *ad libitum*.

### Animal experiment

Following a week of adaptive feeding, STZ-induced type 1 diabetes mellitus (T1DM) mouse model was established. Briefly, mice were injected intraperitoneally with a single dose of 180 mg/kg STZ in 0.1 M sodium citrate buffer. STZ solution was prepared immediately in dark place before use. After 6 h of drug injection, mice were temporarily fed with 10% glucose solution instead of clear water for 12 h to avoid death caused by transient hypoglycemia. After 3 days of drug injection, the fasting blood glucose was measured in each mouse. When the value of fasting blood glucose was greater than 15 mM, these mice were labeled as T1DM for subsequent experiments. Regarding the doses setting of allicin, one previous report showed that the mice received oral treatment with allicin (10 mg/kg/d) for 2 weeks to study the allicin of improving atherosclerosis (Panyod et al., 2022). In addition, the other previous report showed that the mice received oral treatment with allicin (30 mg/kg/d) for 8 weeks to study the allicin of improving the inflammation of diabetic great vessels (Li et al., 2020). Based on these studies, we set the low-dose allicin (10 mg/kg/d) and high-dose allicin (30 mg/kg/d) to treat T1DM mice in this study. The control group was intragastrically administered with an equal volume of clear water. T1DM mice were then received vehicle, Allicin(L) (10 mg/kg) or Allicin(H) (30 mg/kg) by gavage once daily for 6 weeks. Mice were divided into control group (*n* = 10), T1DM group (*n* = 10), T1DM + Allicin(L) group (*n* = 10), and T1DM + Allicin(H) group (*n* = 10). The fasting blood glucose and body weight were measured and recorded weekly. Finally, the serum samples were collected *via* orbital blood sampling and the pancreatic tissues were isolated for subsequent experiments.

### Fasting blood glucose and oral glucose tolerance test

After 12 h of fasting, the blood samples were collected from the tail vein of mice in each group, and blood glucose was measured by glucose meter. In brief, the level of blood glucose in each group after gavage administration with 10% glucose solution (1.0 g/kg) (Li et al., 2008) was measured at different time points, such as 0, 30, 60, 90, and 120 min. Then, OGTT was performed at 1, 3, and 5 weeks after treatment.

### Insulin measurement

The levels of insulin in plasma or cell culture supernatant were measured with mouse insulin ELISA kit according to the manufacturer’s instruction. In brief, both 200 μl samples and 50 μl diluent were added into the pretreated 96-well plates. Then, the plates were incubated for 2 h at room temperature, and rinsed 5 times with washing buffer. 100 μl solution containing peroxidase conjugated IgG anti-insulin antibody was added into wells and incubated for 2 h at room temperature. Then, plates were washed 5 times again with washing buffer. 100 μl substrate buffer was added into each well, and incubated in the dark for 30 min at room temperature. 50 μl stop solution was used to stop the enzyme reaction. The optical concentration was obtained to quantify insulin levels using a microplate reader at the correction wavelength of 450 nm. Data were analyzed by GraphPad Prism 5 software.

### Islet isolation

Islets were isolated as previously described (Xu et al., 2010). Briefly, after ligation of the common bile duct near the liver, the pancreas filled with collagenase V solution (1.0 mg/mL) by retrograde injection through the common bile duct were collected, and then were digested in Hanks balanced salt solution at 37°C for 5–10 min. When most of the pancreatic tissues were digested into chylous and silt, 10 mL pre-chilled Hank’s solution was added to stop digestion, and then the centrifuge was performed after slight oscillation. When the speed reached 2000 rpm, the centrifuge was immediately stopped. The upper layer was discarded, and the precipitates were washed twice with Hank’s solution. 5 mL pre-chilled 1.119 Ficoll was added into the 15 mL centrifuge tube which contained islet tissues and mixed well. Then, 2 mL pre-chilled Ficoll 1.077 solution was gently added to the top, and then 2 mL Hank’s solution was also gently added to the top. After centrifugation at 2000 rpm for 5 min, the precipitates were washed with Hank’s solution again. Then, islets were manually picked out under an anatomical microscope. The isolated islets were immediately used for subsequent experiments.

### Cell culture

The mouse pancreatic β cell line Min6 was gifted by professor Meng from School of Basic Medicine, Zhejiang University, and was cultured in DMEM supplemented with 1% P/S, 15% FBS, 50 μM β mercaptoethanol at a 37°C, 5% CO_2_ incubator.

### Cell experiment

To mimic pancreatic β cell injury model, Min6 was treated with different concentrations of STZ, such as 0.1, 0.2, 0.3, 0.4, 0.5, 0.6, 0.7, and 0.8 mM for 24 h to explore the optimum working concentration. Next, based on the established pancreatic β cell injury model *in vitro*, STZ-induced Min6 was treated with different concentrations of allicin, such as 5, 10, 15, and 20 ng/mL to explore the optimum working concentration. Based on the harvested optimum working concentrations of STZ and allicin, we set different groups including control, T1DM, T1DM + Allicin, and T1DM + Allicin + CC. Cells in T1DM + Allicin group and T1DM + Allicin + CC group were pretreated with 10 ng/mL allicin for 2 h. Then, cells in each group were exposed to the drug for 24 h, and cell culture supernatant or cells were collected for following experiments.

### Cell viability test

CCK-8 was used to analyze the cell viability of Min6 with different treatments. Cells at a density of 2 × 10^3^/well were seeded into 96-well plates. Cells were treated with different concentrations of drug when cell confluency reached 60%–70%. After 24 h of incubation, 110 µl DMEM containing 10 µl CCK-8 reagent was added into each well and incubated at 37°C for 45 min. Lastly, the absorbance at 450 nm was detected with a microplate analyzer (Thermo, MA, United States).

### Histological staining

Mouse pancreas tissues were fixed in 4% paraformaldehyde at 4°C for 24 h. The samples were then dehydrated through graded ethanol and xylene, embedded in paraffin, and cut into 5 μm tissue slices. For histological evaluation, the sections were stained with HE staining kit, and pathological changes in islet tissues were observed under an optical microscope (Nikon, Japan).

### Immunohistochemistry staining

The paraffin sections of pancreas were deparaffinized and washed with xylene twice, and then were rehydrated. The peroxidase blocker was used to cover the entire tissue sections which were later kept at room temperature for 20 min. The sections were then rinsed with PBS, and the antigen was repaired by autoclaving in 10 mM citric acid buffer (pH 6.0). The tissue sections were then blocked with 10% (v/v) goat serum for 1 h at room temperature. Then, these sections were incubated with primary antibodies (listed in Supplementary Table S1) at 4°C for 12 h. Then, they were incubated with goat anti-mouse or anti-rabbit IgG HRP secondary antibody (1:200) for 2 h at room temperature. Finally, they were counterstained with hematoxylin, dehydrated transparent, sealed with neutral resin, and observed under the optical microscope.

### TUNEL staining

TUNEL staining was performed on the pancreatic paraffin sections and cells to detect apoptosis. Briefly, the sections were incubated with protease K for 1 h at 37°C, and washed with PBS. Then, sections were stained with insulin antibody and DAPI. Similarly, the apoptosis of Min6 in each group was detected by immunofluorescence staining with TUNEL reagent. Images were taken under an upright microscope (Nikon ECLIPSE Ti, Japan).

### Real-time quantitative polymerase chain reaction

Total RNAs were extracted from isolated mouse islets or Min6 cells with Trizol reagent. The concentrations of RNAs were measured by reading OD value at 260 nm. These RNAs were reverse-transcribed into cDNA with ReverTra Ace qPCR RT Master Mix, which were used as the templates for RT-qPCR using PrimeScript RT Master Mix reagents. The cycle threshold (Ct) values were obtained and normalized to *GAPDH* levels. Finally, the mRNA expression levels were analyzed with the 2^−ΔΔCt^ method. Primers were listed in Supplementary Table S2.

### Western blot

Protein samples were prepared from isolated mouse islets or Min6 using RIPA lysis buffer supplemented with PMSF. Protein concentrations were determined using a BCA protein assay kit. 20–40 μg proteins were separated by 7.5% or 12.5% SDS-PAGE gels, and then were transferred onto the PVDF membranes. After being blocked by 10% skim milk for 2 h at room temperature, these membranes were then incubated with primary antibodies (listed in Supplementary Table S1) overnight at 4°C, and then incubated with goat anti-mouse or anti-rabbit IgG HRP secondary antibody (1:5000) for 2 h at room temperature. The specific protein bands were finally detected with ECL chromogenic kit, and visualized with ChemiDic ™ XRS imaging system (Bio-Rad, CA, United States). Image Pro Plus 6.1 software was used to analyze the intensity of each gel band.

### Annexin V and PI assay

The Min6 cells from each group were digested with EDTA-free trypsin, and then washed twice with pre-chilled PBS. Then cell apoptosis was detected using Annexin V and PI apoptosis detection commercial kit. Briefly, the harvested cells were resuspended with Binding Buffer, and then were stained with a mixture of 5 µl FITC Annexin V and 5 µl PI in the dark for 15 min at room temperature. The apoptosis ratio was quantified using the Flow Cytometer (Beckman Coulter, CA, United States).

### Immunofluorescence staining

The samples of Min6 cells and pancreatic paraffin sections in different groups were rinsed twice with PBS, and then were fixed with 4% paraformaldehyde for 15 min. Then, the samples were permeabilized with TritonX-100 (0.3%) for 20 min. After washing with PBS again, they were incubated with 10% goat serum for 1 h at room temperature, and then were incubated with primary antibodies (listed in Supplementary Table S1) overnight at 4°C. They were then incubated with FITC or Cy3-conjugated Goat Anti-Rabbit or Mouse IgG secondary antibodies (1:200) for 2 h at room temperature. Afterwards, staining was performed with anti-fluorescence quencher containing DAPI. Subsequently, the samples were stained with DAPI in dark for 15 min at room temperature. Lastly, the samples were visualized by the inverted fluorescence microscope (Leica, Germany).

### Statistical analysis

Data were shown as mean ± standard deviation (SD). All experiments were repeated at least three times. Statistical significance was analyzed by Student’s t-test or one-way ANOVA followed by Turkey’s multiple comparisons. Statistical analysis was implemented using GraphPad Prism version 9 (GraphPad Software Inc., CA, United States). A value of *p* less than 0.05 was considered statistically significant.

## Results

### Allicin improved blood glucose levels and glucose tolerance in T1DM mice

**FIGURE 1:**
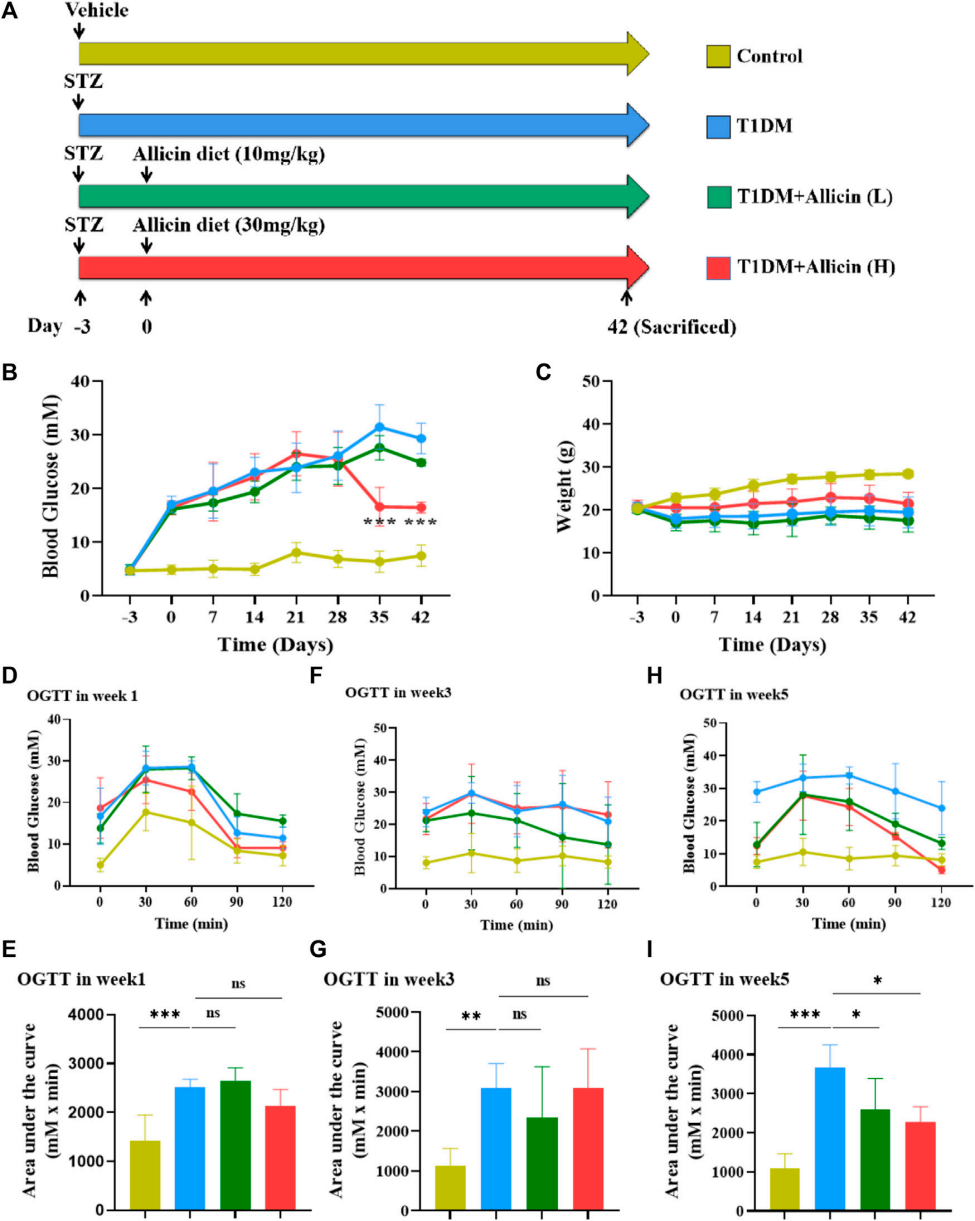
The effects of Allicin on blood glucose levels and glucose tolerance in T1DM mice. **(A)** The schematic diagram depicts the progress of animal experiments with allicin in STZ-induced T1DM mice. Different colors indicate different treatments for mice. **(B)** The fasting blood glucose levels of mice in each group at different time points. **(C)** The body weight of mice in each group at different time points. **(D)** The curve graph of oral glucose tolerance test (OGTT) from 0 to 120 min at week 1. **(E)** The quantitative results of OGTT at week 1. **(F)** The curve graph of OGTT from 0 to 120 min at week 3. **(G)** The quantitative results of OGTT at week 3. **(H)** The curve graph of OGTT from 0 to 120 min at week 5. **(I)** The quantitative results of OGTT at week 5. *N* = 3, Mean ± SD. ^*^ *p* < .05, ^**^ *p* < .01, ^***^ *p* < .001 indicate significant differences, and ns > .05 means no significance difference.

To evaluate the protective effects of allicin on pancreatic β cells in type 1 diabetes *in vivo*, STZ-induced T1DM mouse model was established and subjected to different treatments as shown in Figure 1A. Hyperglycemia is an important character of T1DM. To investigate the effects of allicin on STZ-induced T1DM, the levels of fasting blood glucose in different groups were measured weekly for 6 weeks. The results showed that the level of blood glucose in control group was stabilized at < 15 mM, and the level of blood glucose in T1DM group was increased gradually at ≥ 15 mM over time and reached the highest on day 35. Allicin (L) treatment could partially decrease the blood glucose level in STZ-induced T1DM mice, but had no significant difference compared with T1DM group. Allicin (H) administration could significantly reduce blood glucose level from day 35–42 in STZ-induced T1DM mice (Figure 1B). The body weight in each group kept increasing gradually from day 0 to day 28, and the body weight in STZ-induced T1DM mice with Allicin (L) and Allicin(H) treatments became decreasing except for control and T1DM groups from day 28 to day 42. Allicin (H) treatment could partially inhibit the decrease of body weight in STZ-induced T1DM mice, but had no significant difference compared with T1DM group (Figure 1C). In addition, the results of OGTT showed that the blood glucose levels from 0 to 120 min at week 1, 3, and 5 in T1DM group were higher than that of control group, and the quantitative data of OGTT displayed that glucose tolerance was significantly impaired compared with control group. Both Allicin (L) and Allicin (H) treatments could improve glucose tolerances at week 1, 3, and 5 in STZ-induced T1DM mice, but had no significant differences compared with T1DM group except for week 5 (Figures 1D–I). These results suggested that allicin could improve hyperglycemia of STZ-induced T1DM mice.

### Allicin improved the islet morphology and pancreatic β cell function in T1DM mice

**FIGURE 2:**
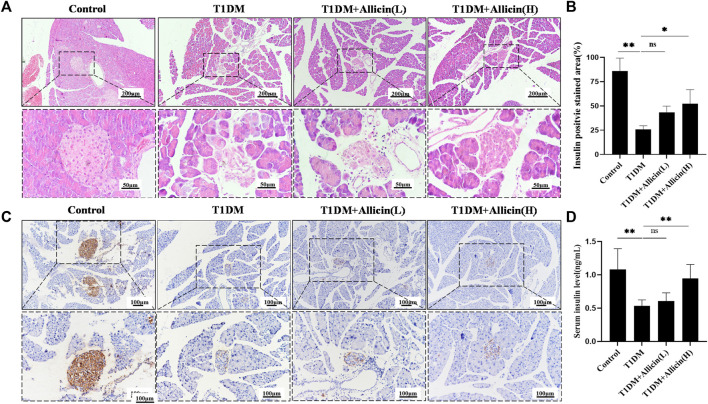
Allicin attenuated the islet structure and function in T1DM mice. **(A)** Hematoxylin-Eosin (HE) staining of pancreatic sections in each group. **(B)** The immumohistochemical staining of insulin in each group. **(C)** The quantitative analysis of immumohistochemical staining (*n* = 3). **(D)** ELISA analysis of serum fasting insulin levels (*n* = 3). Mean ± SD. ^*^ *p* < .05, ^**^ *p* < .01, ^***^ *p* < .001 indicate significant differences, and ns > .05 shows no difference. Scale bars 200, 100, and 50 μm.

HE staining was performed to assess the changes of pathological morphological structure of pancreatic islet tissues in each group. The result showed that the morphology of islet in control group was normal, having tight arrangement, abundant cytoplasm, and clear nucleolus of pancreatic β cells. However, the islet in T1DM group was small and disordered with inconspicuous boundary, irregular shape, vacuolated degeneration, and cytolysis of pancreatic β cells. Both Allicin (L) and Allicin (H) treatments could partially rescue the impaired islet morphology of STZ-induced T1DM mice, and Allicin (H) had the best effects (Figure 2A). Meanwhile, the level of serum insulin in T1DM group was significantly decreased compared with control group, and Allicin (L) or Allicin (H) administration could recover the serum insulin level in STZ-induced T1DM mice, while Allicin (L) treatment had no significant difference compared with T1DM group (Figure 2B). In addition, the result of immumohistochemical staining of INSULIN was almost consistent with HE staining (Figure 2C). The quantitative result showed that, compared with T1DM group, Allicin (H) administration could significantly increase the expression of INSULIN in STZ-induced T1DM mice (Figure 2D).

**FIGURE 3:**
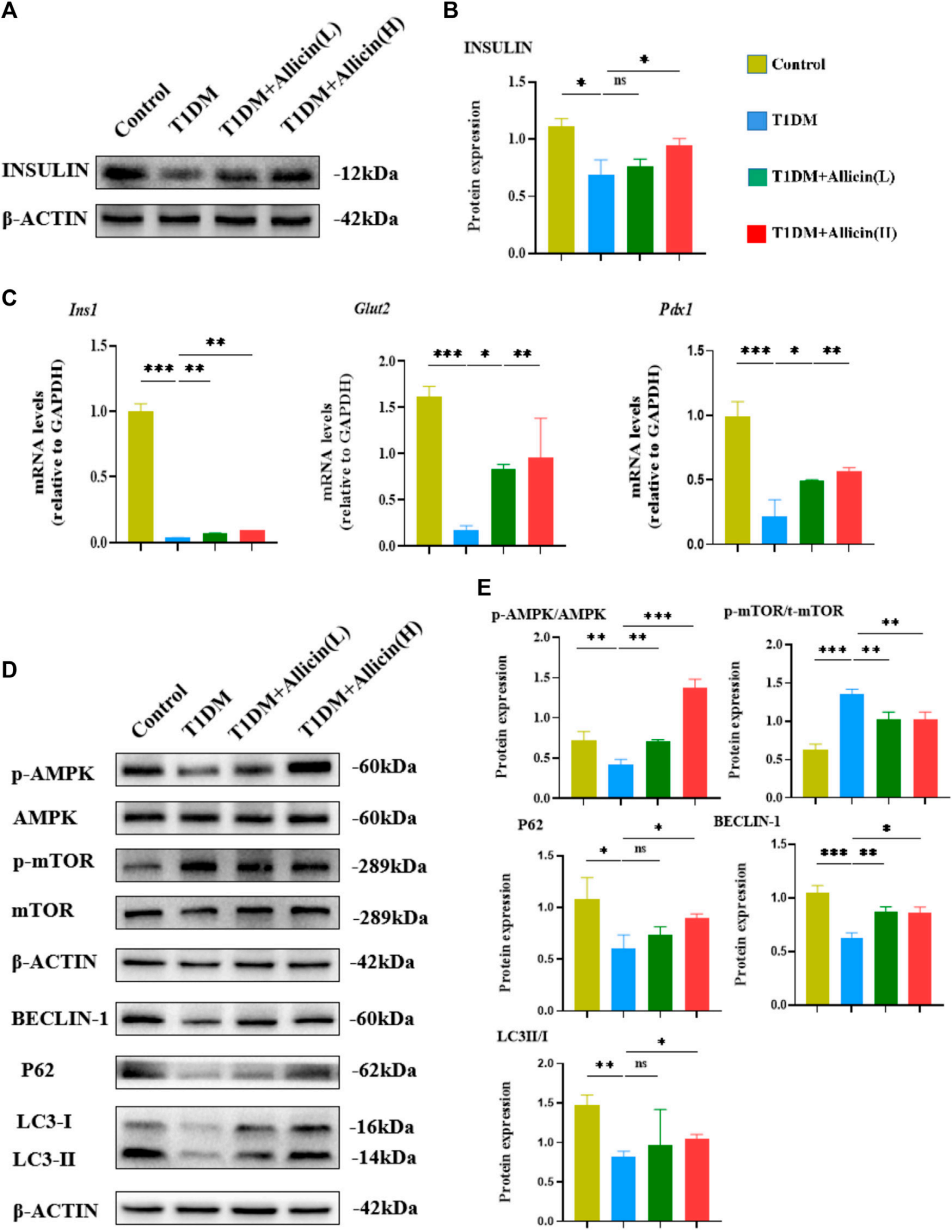
Allicin mediated pancreatic β cell autophagy through activating AMPK/mTOR pathway in T1DM mice. **(A)** Western blot gel images of INSULIN and β-ACTIN in each group. **(B)** The bar graph represents the quantification of gel bands. **(C)** The mRNA levels of *Ins1*, *Glut2*, and *Pdx1* in each group by RT-qPCR (*n* = 3). **(D)** The western blot gel images of proteins in AMPK/mTOR-mediated autophagy flux pathway in each group. **(E)** The bar graphs represent the quantification of gel bands (*n* = 3). Mean ± SD. ^*^ *p* < .05, ^**^ *p* < .01, ^***^ *p* < .001 indicate significant differences, and ns > .05 shows no difference.

Moreover, western blot was used to detect the expression of INSULIN protein in different groups (Figure 3A), and the quantitative result was consistent with the above serum insulin result (Figure 3B). The results of RT-qPCR showed that the levels of islet function-related genes, such as *Ins1*, *Glut2*, and *Pdx1* were significantly lowered compared with control group, and Allicin (L) or Allicin (H) administration could significantly up-regulate these gene levels in STZ-induced T1DM mice (Figure 3C). These data suggested that allicin could improve the islet morphology and pancreatic β cell function for insulin production in STZ-induced T1DM mice.

### Allicin activated the AMPK/mTOR mediated autophagy pathway in T1DM mice

Previous studies had demonstrated that autophagy could be involved in T1DM (Gonzalez et al., 2011; Darwish et al., 2021). AMPK acts as an upstream factor of mTOR, which can participate in the regulation of autophagy, and AMPK activation has an antagonistic effect on mTOR (Zhai et al., 2018). Therefore, the AMPK/mTOR-mediated autophagy pathway was detected to assess the protective effects of allicin on pancreatic β cells in T1DM mice by western blot (Figure 3D). The results showed that the levels of autophagy-related proteins, such as LC3-II/I, BECLIN-1, P62 as well as p-AMPK/AMPK were down-regulated and p-mTOR/mTOR was up-regulated in T1DM group compared with control group. Allicin (L) and Allicin (H) treatments could almost reverse this trend in T1DM mice (Figure 3E). These results suggested that allicin might protect pancreatic β cells of STZ-induced T1DM mice through activating the AMPK/mTOR mediated autophagy pathway.

### Allicin attenuated the apoptosis of pancreatic β cells in T1DM mice

**FIGURE 4:**
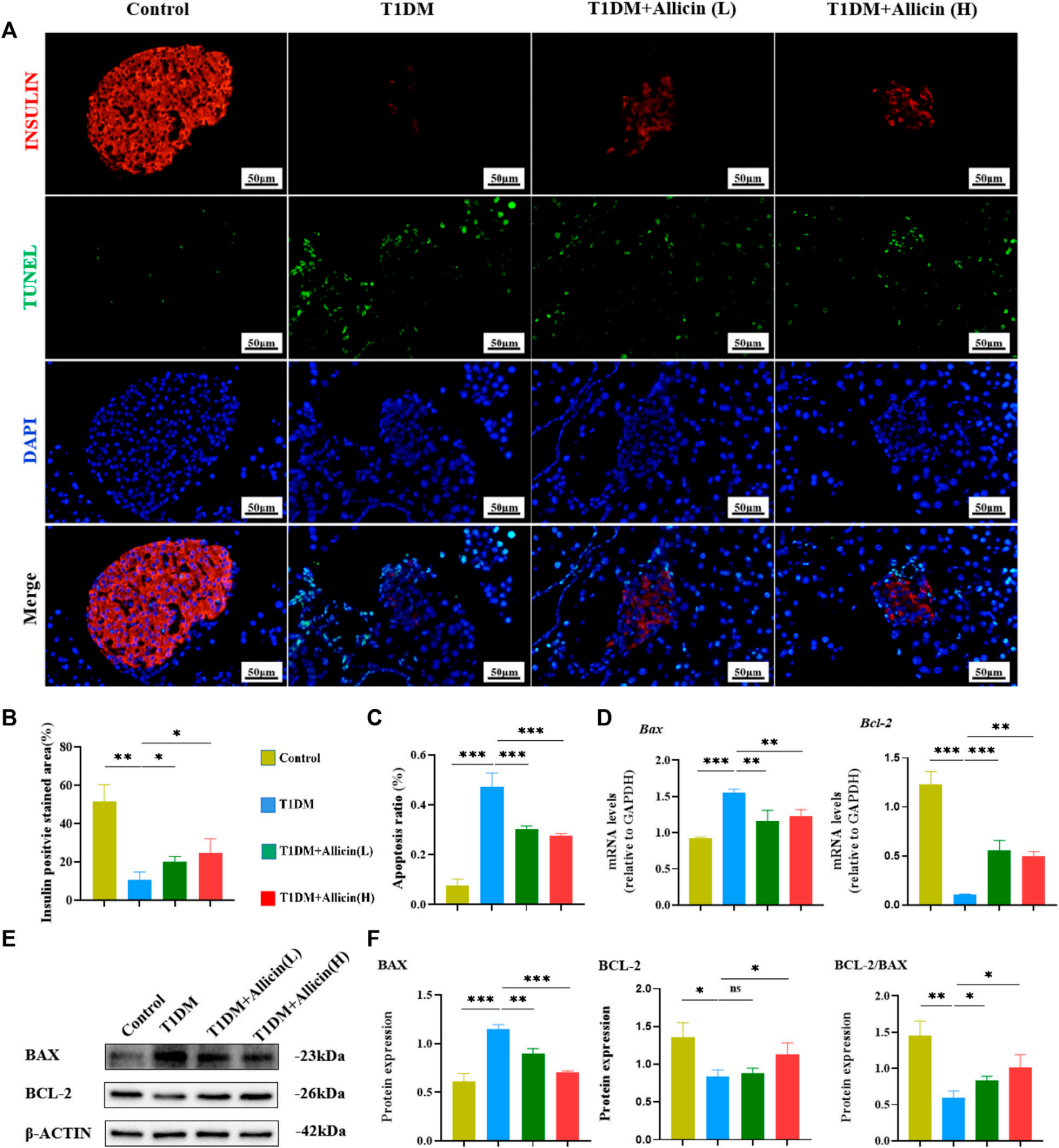
Allicin attenuated the apoptosis of pancreatic β cells in T1DM mice. **(A)** The exploration of pancreatic β cell apoptosis using TUNEL staining. The red staining represents insulin secreted by pancreatic β cells. The green staining represents apoptosis. **(B)** The quantification results of insulin staining in each group (*n* = 3). **(C)** The quantification results of TUNEL staining in different groups (*n* = 3). **(D)** The mRNA level of *Bax* and *Bcl-2* in each group by RT-qPCR (*n* = 3). **(E)** Western blot gel images of BAX and BCL-2 in each group. **(F)** The bar graphs represent the quantification of western blot gel bands (*n* = 3) and the ratio of BCL-2 and BAX proteins. Mean ± SD. ^*^ *p* < .05, ^**^ *p* < .01, ^***^ *p* < .001 indicate significant differences, and ns > .05 shows no difference. Scale bars 50 μm.

Pancreatic β cells, as the most important component in islet tissues, are the only functional cells for insulin secretion *in vivo*. Homeostasis of the number and the function of pancreatic β cells are really essential for blood glucose regulation. The apoptosis of pancreatic β cells is considered to be an important cause for the development of T1DM (Kim et al., 1999). The TUNEL combined with INSULIN co-staining were performed to detect the apoptosis of pancreatic β cells in STZ-induced T1DM mice (Figure 4A). The results showed that the INSULIN-positive cells almost disappeared, and the number of TUNEL-positive cells were significantly increased in T1DM group compared with control group. Allicin (L) and Allicin (H) treatments could increase the number of INSULIN-positive cells (Figure 4B) and decrease the number of TUNEL-positive cells in STZ-induced T1DM mice (Figure 4C). In addition, the results of RT-qPCR showed that compared with control group, the expression of apoptosis gene *Bax* was up-regulated and the level of anti-apoptosis gene *Bcl-2* was down-regulated in T1DM group. Both Allicin (L) and Allicin (H) could significantly reverse this trend in STZ-induced T1DM mice (Figure 4D). Moreover, the expressions of apoptosis protein BAX and anti-apoptosis BCL-2 were detected by western blot (Figure 4E). The quantitative results showed that the apoptosis protein BAX was significantly up-regulated, and the anti-apoptosis protein BCL-2 as well as the level of BCL-2/BAX were significantly down-regulated in T1DM group compared with control group. Allicin (L) and Allicin (H) treatments could significantly reverse this trend, except that BCL-2 in Allicin (L) had no statistical significance in STZ-induced T1DM mice (Figure 4F). These results suggested that allicin could ameliorate pancreatic β cell apoptosis in T1DM mice.

### Detect the optimal working concentrations of STZ and allicin on pancreatic β cells

We employed STZ to induce Min6 to mimic type 1 diabetic condition *in vitro*. Initially, we set different concentrations of STZ ranging from 0 to 0.8 mM to treat Min6. The results of CCK-8 revealed that the concentrations of STZ at 0.4 mM could greatly restrain the cell viability of Min6 (Supplementary Figure S1A). Therefore, the following working concentration of STZ was set at 0.4 mM to induce Min6 to establish T1DM cell model *in vitro*. In addition, we set diverse concentrations of allicin ranging from 5 to 20 ng/mL to treat STZ-induced Min6. The results of CCK-8 showed that the concentrations of allicin at 10 ng/mL could greatly improve the cell viability of STZ induced Min6 (Supplementary Figure S1B). So, we selected 10 ng/mL as the following working concentration of allicin to treat Min6.

### Allicin improved pancreatic β cell function *in vitro*

**FIGURE 5:**
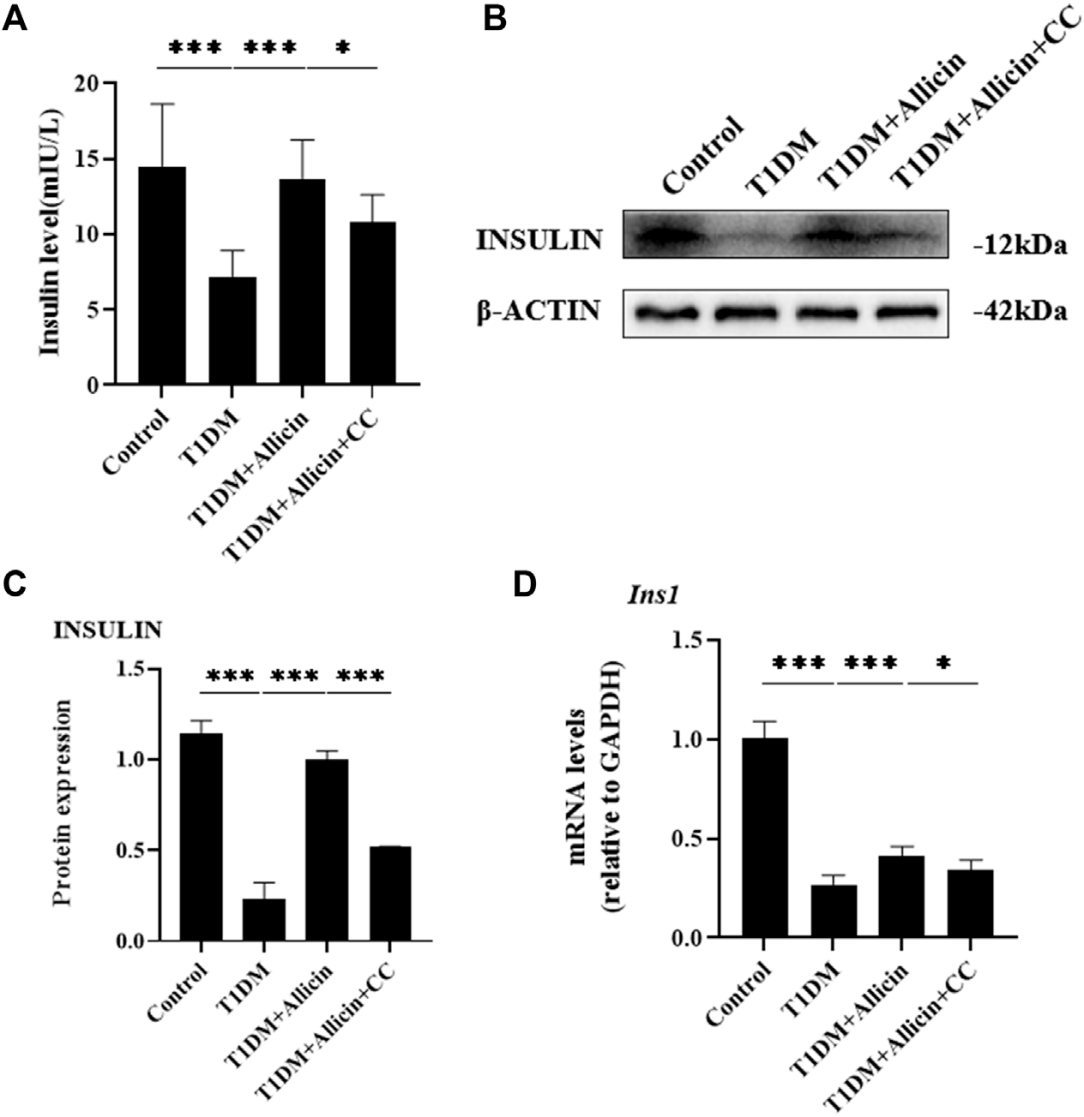
Allicin improved the insulin secretion function of STZ-induced pancreatic β cell Min6 *in vitro*. **(A)** ELISA analysis of the insulin levels in the cell culture supernatant from each group. **(B)** Western blot gel images of INSULIN and β-ACTIN in each group. **(C)** The bar graphs represent the quantification of INSULIN protein levels. **(D)** The mRNA levels of *Ins1* in each group by RT-qPCR. *N* = 3, Mean ± SD. ^*^ *p* < .05, ^**^ *p* < .01, ^***^ *p* < .001 indicate significant differences.

To assess the protective function of allicin on STZ-induced pancreatic β cell Min6 *in vitro*, the cell culture supernatants in different groups were harvested for insulin level measurement using ELISA. The results showed that the level of insulin in T1DM group was significantly lower than that of control group. After allicin treatment, the level of insulin was significantly increased in STZ-induced Min6, which could be markedly reversed by AMPK inhibitor CC (Figure 5A). In addition, we detected insulin gene or protein expression in each group by western blot (Figure 5B) and RT-qPCR, and the results were consistent with the above ELISA data (Figures 5C,D). These results further suggested that allicin could resume the insulin secretion of STZ-induced pancreatic β cell Min6.

### Allicin inhibited the apoptosis of STZ-induced pancreatic β cells *in vitro*

**FIGURE 6:**
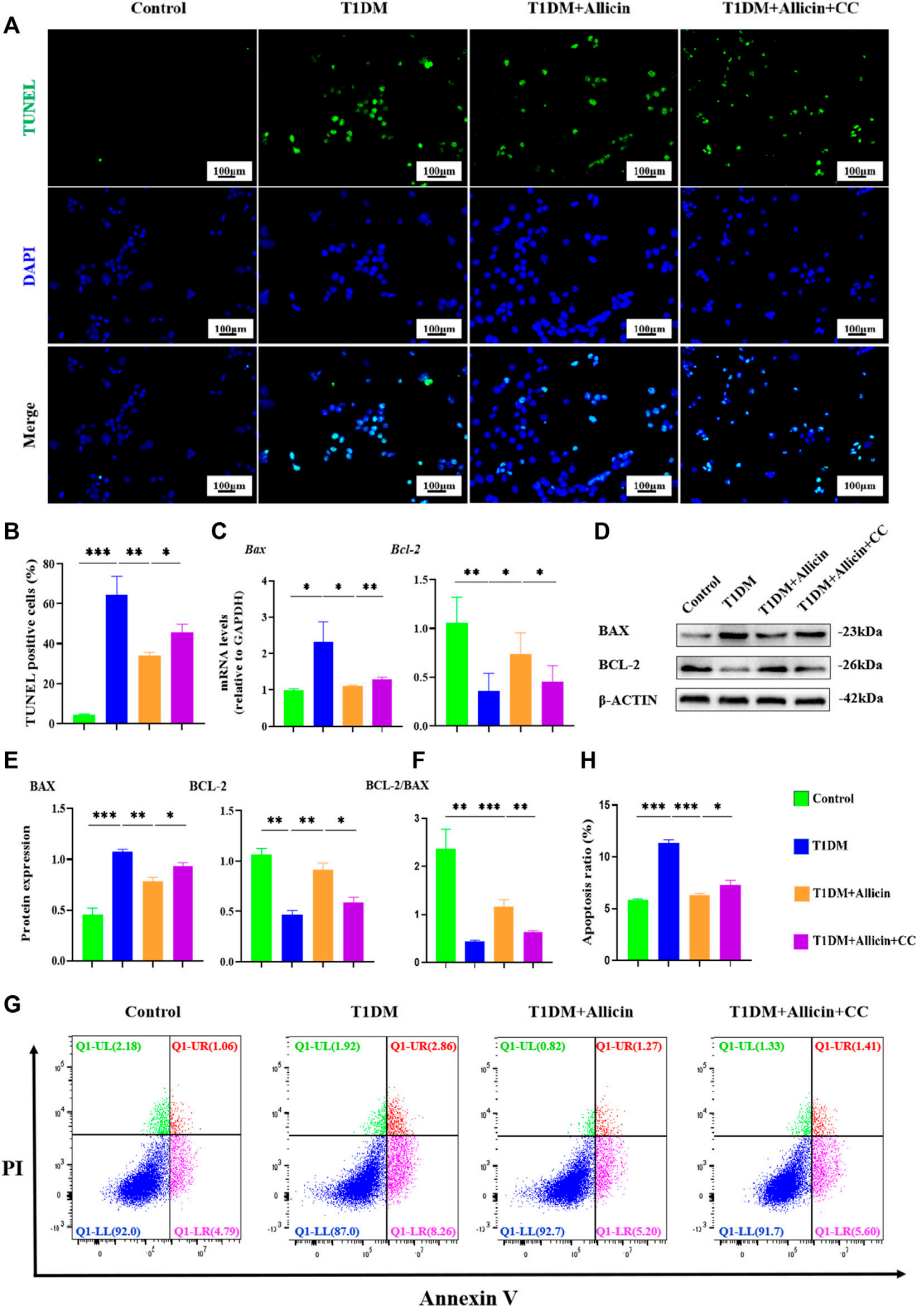
Allicin attenuated the apoptosis of STZ-induced pancreatic β cell Min6 *in vitro*. **(A)** The detection of pancreatic β cells apoptosis in each group using TUNEL staining. The cells with green fluorescence represents apoptosis. **(B)** The quantification of TUNEL staining. **(C)** The mRNA level of *Bax* and *Bcl-2* in each group by RT-qPCR. **(D)** Western blot gel images of BAX and BCL-2 in each group. **(E)** The bar graphs represent the quantification of protein levels. **(F)** The ratio of BCL-2 and BAX proteins. **(G)** The exploration of the cell apoptosis using Annexin V-PI apoptosis detection kit by flow cytometry. **(H)** The quantitation of flow cytometry assay. N = 3, Mean ± SD. ^*^ *p* < .05, ^**^ *p* < .01, ^***^ *p* < .001 indicate significant differences. Scale bars 100 μm.

To further investigate the anti-apoptosis effect of allicin on STZ-induced pancreatic β cell Min6 *in vitro*, TUNEL staining was conducted to detect the apoptosis in each group (Figure 6A). The result of TUNEL staining showed that the number of TUNEL-positive cells was significantly increased in T1DM group compared with control group. Allicin treatment could significantly decrease TUNEL-positive cells in STZ-induced Min6, and AMPK inhibitor CC could abolish the effect of allicin (Figure 6B). Meanwhile, the RT-qPCR result showed that the level of apoptosis gene *Bax* was up-regulated and the expression of anti-apoptosis gene *Bcl-2* was down-regulated in T1DM group, and allicin treatment could reverse this trend in STZ-induced Min6, while AMPK inhibitor CC could abrogate the effect of allicin (Figure 6C). In addition, the expression of apoptosis protein BAX and anti-apoptotic protein BCL-2 in each group was detected by western blot (Figure 6D), and the quantitative result was almost consistent with the above RT-qPCR result (Figure 6E). Moreover, the flow cytometry of Annexin V-PI assay was used to detect the cell apoptosis in each group (Figure 6F). The percentages of apoptosis cells are calculated from the early apoptosis (Q1-LR) and late apoptosis (Q1-UR). The quantitative results were consistent with the above TUNEL staining results (Figure 6G). These data further suggested that allicin could ameliorate STZ-induced pancreatic β cell apoptosis.

### Allicin protected pancreatic β cells *in vitro* through activating AMPK/mTOR mediated autophagy pathway

**FIGURE 7:**
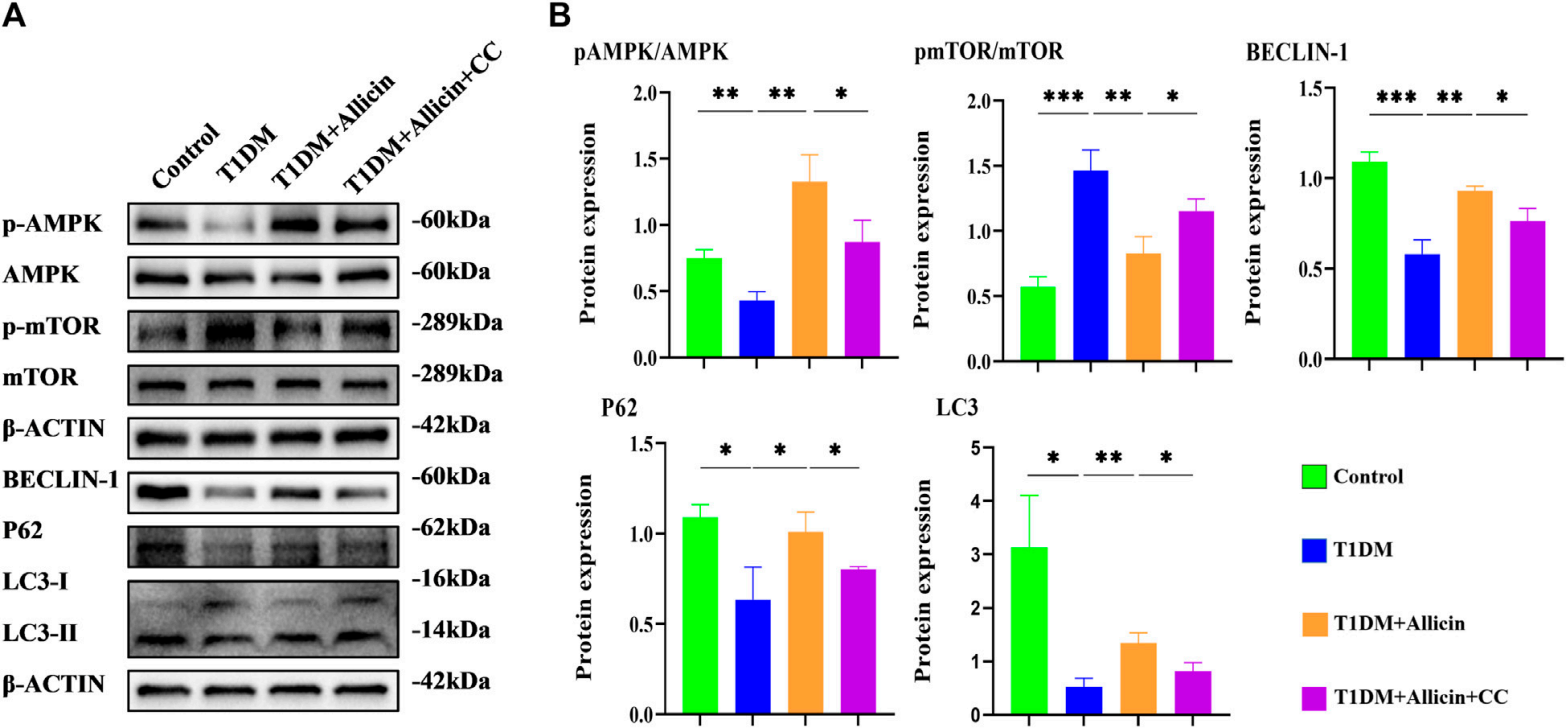
Allicin mediated pancreatic β cell Min6 autophagy through activating AMPK/mTOR *in vitro*. **(A)** Western blot gel images of proteins in AMPK/mTOR-mediated autophagy flux pathway *in vitro*. **(B)** The bar graphs represent the quantification of protein levels in each group (*n* = 3). Mean ± SD. ^*^ *p* < .05, ^**^ *p* < .01, ^***^ *p* < .001 indicate significant differences.

**FIGURE 8:**
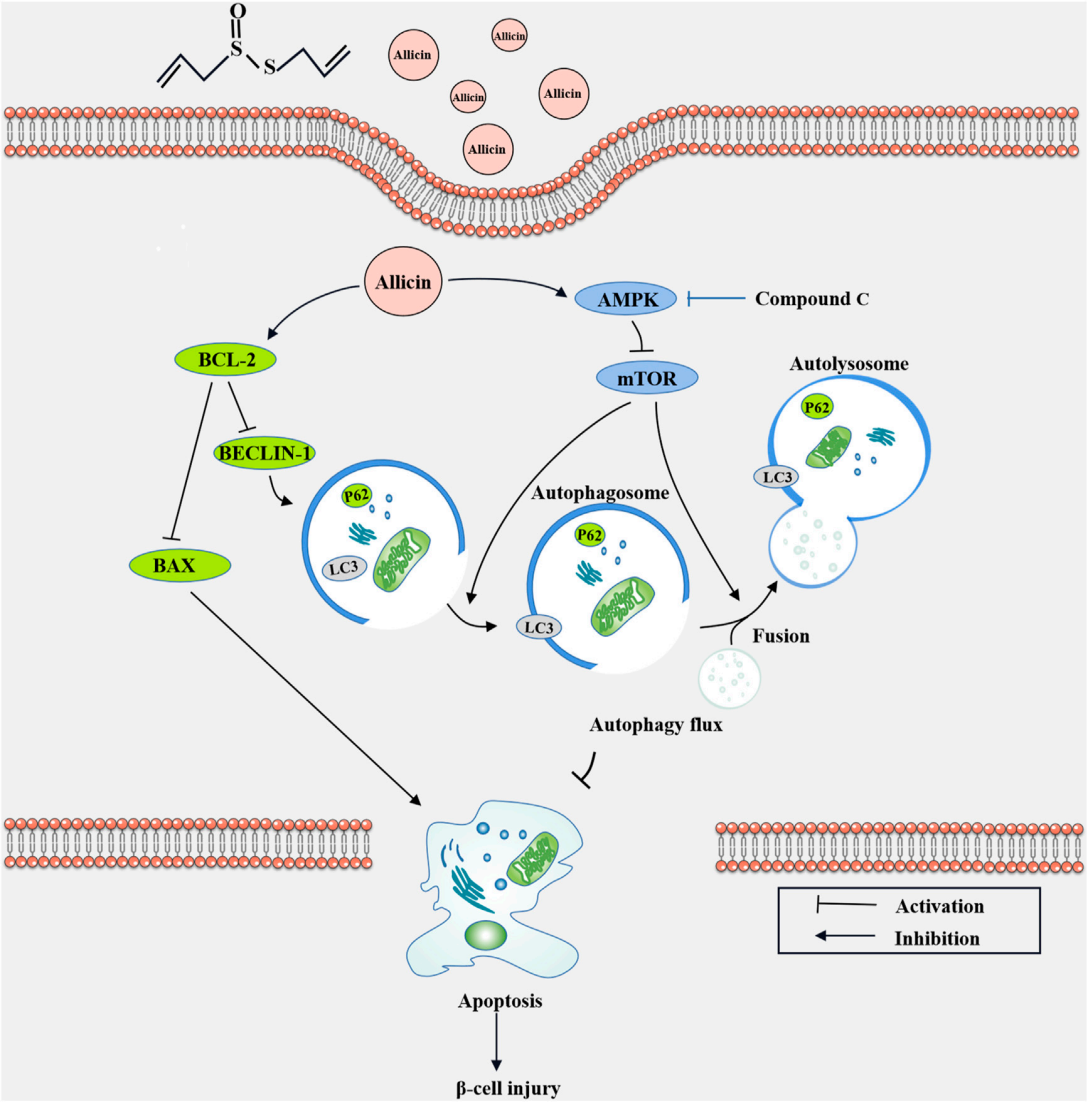
The diagram of the underlying mechanism for Allicin protection roles in T1DM through AMPK/mTOR mediated autophagy pathway.

To further explore the potential protective mechanism of allicin on pancreatic β cells, western blot was used to detect the AMPK/mTOR-mediated autophagy pathway to assess the protective effects of allicin on pancreatic β cells in STZ-induced Min6 *in vitro* (Figure 7A). The results of western blot showed that the levels of p-AMPK/AMPK, LC3-II/I, BECLIN-1, and P62 were significantly down-regulated and p-mTOR/mTOR was observably up-regulated in T1DM group compared with control group. Allicin administration could reverse this trend in STZ-induced Min6, but AMPK inhibitor CC could abolish the effect of allicin (Figure 7B). In addition, the immunofluorescence staining of LC3 (Supplementary Figure S2A) and P62 (Supplementary Figure S2C) were conducted in different groups, and the quantitative results show that the trend of LC3 (Supplementary Figure S2B) and P62 (Supplementary Figure S2D) expressions was consistent with western blot results. Based on the above data, we could speculate that allicin might play the protective roles on pancreatic β cells of STZ-induced T1DM through activating the AMPK/mTOR mediated autophagy pathway. The detailed mechanism diagram is shown in Figure 8.

## Discussion

Type 1 diabetes mellitus (T1DM) is a medical problem that imposes a huge economic burden on society worldwide. T1DM occurs frequently in adolescents, which is characterized by high blood glucose levels, and is an autoimmune-mediated chronic disease. The etiology of T1DM is very complex, involving the auto-antibody targeting the islets of Langerhans (Krischer et al., 2015), genetics (Tuomilehto, 2013), and environmental factors (Ashton et al., 2016; Hyoty, 2016). All of those factors eventually lead to an absolute lack of insulin secretion in pancreatic β cells and make the treatment options very scarce. Insulin injection is currently the only way to treat T1DM. However, this therapy has some limitations and can cause complications, such as fatal hypoglycemia, diabetic ketoacidosis, and a series of microangiopathies (Katsarou et al., 2017). Therefore, it is particularly necessary to develop more therapy drugs.

The use of natural extracts from plants in regulating the blood sugar and lipid levels and preventing diabetes complications has attracted much attention. Allicin, a sulfur-containing compound with strong biological activity in garlic, has the therapeutic effects on diabetic nephropathy, diabetic wound healing, and other diseases (Osman et al., 2012; Arellano Buendia et al., 2018; Toygar et al., 2020). In the present study, we investigated the effects of allicin on T1DM, and further revealed the underlying mechanism.

Pancreatic β cells are involved in the progression of T1DM, so the therapy targeting pancreatic β cell injury is an ideal strategy for T1DM treatment and its complications. STZ, a spectral antibiotic that contains a highly active methyl-nitrourea part in its structure, is a classical T1DM modeling drug (Lenzen, 2008), which can bind to glucose molecules and play cytotoxic effects (Vavra et al., 1959). Therefore, in this study, we established the STZ-induced mouse model *in vivo* and STZ-induced Min6 cell model *in vitro* to explore the effects of allicin on T1DM. The results showed that the fasting blood glucose level of T1DM mice always indicated hyperglycemia, while the high dose allicin could significantly decrease the fasting blood glucose level in STZ-induced T1DM mice after 6 weeks of continuous treatment. In addition, in order to understand the carbohydrate loading capacity, as well as the changes of dynamic blood glucose levels and the numbers of pancreatic β cells in islets, the oral glucose tolerance test (OGTT) and fasting insulin detection were performed. The results showed that allicin could significantly improve the oral glucose tolerance and fasting insulin secretion in week five and week six in STZ-induced T1DM mice. These findings suggested that allicin has certain therapeutic effects on T1DM.

Human pancreatic β cells are the only insulin-producing cells in the body and play a crucial role in maintaining glucose homeostasis. Theoretically, the number of pancreatic β cells is kept constant, but the recent evidence documented that the number of pancreatic β cells was dynamically regulated to change throughout life (Ding et al., 2013). Autophagy can be involved in glucose metabolism diseases, and the recent study showed that cannabinoid could play an anti-inflammatory role to treat type 1 diabetes through autophagy pathways (Liu et al., 2021). The cPKCγ knock-out could alleviate the cognitive dysfunction caused by type 1 diabetes through AMPK/mTOR mediated autophagy pathway (Zheng et al., 2022). We suspected that the therapy focused on potential cytoprotective effects of autophagy in diabetes might be a promising solution. Autophagy-lysosome pathway, a conserved proteolytic mechanism, is that the damaged or dysfunctional intracellular cytoplasmic components are transported into lysosomes for breakdown (Yang and Klionsky, 2010; Mihalache and Simon, 2012; Wirawan et al., 2012). LC3 is an autophagy biomarker that plays an important role in the formation of autophagosomes (Notte et al., 2011). When autophagy is formed, the cytosolic-type LC3 (LC3-I) will decompose a small piece of polypeptide to transform into autophagosome membrane-type LC3 (LC3-II), and the ratio of LC3-II/I can indirectly reflect autophagy (Zois and Koukourakis, 2009). BECLIN-1 is also required for autophagy, and when phosphorylated, BECLIN-1 can dissociate from BCL-2 to initiate autophagy (Yue et al., 2003; Yuan et al., 2012). P62, a multifunctional ubiquitin-binding protein which can bind to LC3, will be wrapped by autophagosomes and enter into lysosomes for degradation (Mizushima et al., 2010; Lu and Hu, 2016). In our study, allicin could upregulate the levels of LC3II/I and BECLIN-1 in STZ-induced mouse or cell models, suggesting activation of autophagy. Interestingly, the expression of P62 was also elevated by allicin, which might be due to compensatory increases in the numbers of autophagosome and autolysosome rather than blockage of autophagy (Zheng et al., 2011).

AMPK is a sensor of the overall energy charge of a cell, which can regulate cellular metabolism to maintain energy homeostasis and participate in autophagy (Marwick, 1993; Bischoff et al., 2012). mTOR, as a downstream of AMPK, can be also involved in the regulation of autophagy. In this study, allicin could activate AMPK/mTOR pathway in STZ-induced mouse and cell models through increasing pAMPK/AMPK ratio and decreasing pmTOR/mTOR ratio. In addition, AMPK inhibitor compound C (CC) could significantly reverse the effects of allicin. These data suggested that allicin could activate the AMPK/mTOR mediated autophagy to play a protective role against STZ-induced pancreatic β cell injury.

By the time of T1DM diagnosis, there are about 70%–80% of the β-cell masses injured or even lost because of β-cell apoptosis (Ding et al., 2013). Pancreatic β-cell apoptosis is indispensable for the pathogenesis of T1DM, and autophagy is an important regulator of apoptosis (Degenhardt et al., 2006). The relationship between autophagy and apoptosis is complex (Nagata, 1997), and the autophagy can be manifested as stress adaptation to inhibit apoptosis or prevent cell death (Adrain and Martin, 2001). BAX is a pro-apoptotic cytoplasmic protein of the BCL-2 family, and BAX and BCL-2 are the main mediators of endogenous apoptosis. Apoptosis can be activated by apoptotic protein BAX and inhibited by anti-apoptotic protein BCL-2 (Wang et al., 2015). In our study, allicin could significantly down-regulate the expression of BAX and up-regulate the level of BCL-2 in STZ-induced mouse or cell models. However, AMPK inhibitor CC could offset these effects of allicin. Meanwhile, the results of TUNEL staining and flow cytometry were consistent with this trend, suggesting that allicin could alleviate STZ-induced apoptosis of pancreatic β cells through AMPK/mTOR pathway. Certainly, there are still some limitations in our study. T1DM involves autoimmune-mediated destruction of pancreatic β cells, and the pathological process of T1DM may involve survival/death of immune cells (English et al., 2009). In this study, the specific roles of allicin on immune cells in T1DM were not explored. Moreover, only AMPK inhibitor was used in present study, and mTOR inhibitor has not been tested.

In conclusion, our study demonstrated that allicin could significantly alleviate STZ-induced pancreatic β cells injury *in vivo* and *in vitro* T1DM models. Allicin could significantly decrease blood glucose level, improve islet structure and insulin expression, and inhibit apoptosis to reduce STZ-induced pancreatic β cell injury and loss through activating AMPK/mTOR mediated autophagy pathway. In summary, our study showed that allicin treatment could significantly reduce STZ-induced T1DM progression, suggesting that allicin may be a potential therapy option for T1DM patients.

## Data Availability

The original contributions presented in the study are included in the article/Supplementary Materials, further inquiries can be directed to the corresponding authors.

## Supplementary material

Supplementary Figure S1 The optimal working concentrations of STZ and Allicin on pancreatic β cell Min6 *in vitro*. **(A)** The effects of STZ on cell viability of pancreatic β cell Min6 in each group by CKK-8 assay (*n*=5). **(B)** The effects of allicin on cell viability of pancreatic β cell Min6 in each group by CKK-8 assay (*n*=5). Mean ± SD. ^***^ *p* < 0.05, ^*****^ *p* < .001 indicate significant differences.

Supplementary Figure S2 Immunofluorescence staining for LC3 and P62 in pancreatic β cell Min6 *in vitro*. **(A)** Immunofluorescence staining for LC3 in pancreatic β cell Min6 from each group. The green staining designates LC3, and the blue staining shows the nucleus. **(B)** The bar graph represents the quantification of protein levels in each group (*n* = 3). **(C)** Immunofluorescence staining for P62 in pancreatic β cell Min6 from each group. The green staining designates P62, and the blue staining shows the nucleus. **(D)** The bar graph represents the quantification of protein levels in each group (*n* = 3). Mean ± SD. ^***^ *p* < .05, ^*****^ *p* < .001 indicate significant differences. Scale bars 100 μm.

## References

[B1] Adrain C. Martin S. J. (2001). The mitochondrial apoptosome: A killer unleashed by the cytochrome seas. Trends Biochem. Sci. 26, 390–397. 10.1016/s0968-0004(0101844-8) 11406413

[B2] Arellano Buendia A. S. Tostado Gonzalez M. Sanchez Reyes O. Garcia Arroyo F. E. Arguello Garcia R. Tapia E. (2018). Immunomodulatory effects of the nutraceutical garlic derivative allicin in the progression of diabetic nephropathy. Int. J. Mol. Sci. 19 (10), 3107. 10.3390/ijms19103107 30314265 PMC6212798

[B3] Ashton M. P. Eugster A. Walther D. Daehling N. Riethausen S. Kuehn D. (2016). Incomplete immune response to coxsackie B viruses associates with early autoimmunity against insulin. Sci. Rep. 6, 32899. 10.1038/srep32899 27604323 PMC5015062

[B4] Atkinson M. A. Eisenbarth G. S. Michels A. W. (2014). Type 1 diabetes. Lancet 383 (9911), 69–82. 10.1016/S0140-6736(13)60591-7 23890997 PMC4380133

[B5] Ba L. Gao J. Chen Y. Qi H. Dong C. Pan H. (2019). Allicin attenuates pathological cardiac hypertrophy by inhibiting autophagy via activation of PI3K/Akt/mTOR and MAPK/ERK/mTOR signaling pathways. Phytomedicine 58, 152765. 10.1016/j.phymed.2018.11.025 31005720

[B6] Bischoff P. Josset E. Dumont F. J. (2012). Novel pharmacological modulators of autophagy and therapeutic prospects. Expert Opin. Ther. Pat. 22 (9), 1053–1079. 10.1517/13543776.2012.715148 22860892

[B7] Borlinghaus J. Albrecht F. Gruhlke M. C. Nwachukwu I. D. Slusarenko A. J. (2014). Allicin: Chemistry and biological properties. Molecules 19 (8), 12591–12618. 10.3390/molecules190812591 25153873 PMC6271412

[B8] Capasso A. (2013). Antioxidant action and therapeutic efficacy of Allium sativum L. Molecules 18 (1), 690–700. 10.3390/molecules18010690 23292331 PMC6269925

[B9] Cheng C. I. Lee Y. H. Chen P. H. Lin Y. C. Chou M. H. Kao Y. H. (2017). Cobalt chloride induces RhoA/ROCK activation and remodeling effect in H9c2 cardiomyoblasts: Involvement of PI3K/Akt and MAPK pathways. Cell Signal 36, 25–33. 10.1016/j.cellsig.2017.04.013 28435089

[B10] Darwish M. A. Abdel-Bakky M. S. Messiha B. A. S. Abo-Saif A. A. Abo-Youssef A. M. (2021). Resveratrol mitigates pancreatic tf activation and autophagy-mediated beta cell death via inhibition of CXCL16/ox-LDL pathway: A novel protective mechanism against type 1 diabetes mellitus in mice. Eur. J. Pharmacol. 901, 174059. 10.1016/j.ejphar.2021.174059 33794215

[B11] Degenhardt K. Mathew R. Beaudoin B. Bray K. Anderson D. Chen G. (2006). Autophagy promotes tumor cell survival and restricts necrosis, inflammation, and tumorigenesis. Cancer Cell 10 (1), 51–64. 10.1016/j.ccr.2006.06.001 16843265 PMC2857533

[B12] Ding L. Gysemans C. Mathieu C. (2013). β-Cell differentiation and regeneration in type 1 diabetes. Diabetes Obes. Metab. 15 (3), 98–104. 10.1111/dom.12164 24003926

[B13] English L. Chemali M. Duron J. Rondeau C. Laplante A. Gingras D. (2009). Autophagy enhances the presentation of endogenous viral antigens on MHC class I molecules during HSV-1 infection. Nat. Immunol. 10 (5), 480–487. 10.1038/ni.1720 19305394 PMC3885169

[B14] Gao X. Chen Y. Chen Z. Xue Z. Jia Y. Guo Q. (2019). Identification and antimicrobial activity evaluation of three peptides from laba garlic and the related mechanism. Food Funct. 10 (8), 4486–4496. 10.1039/c9fo00236g 31241636

[B15] Gonzalez C. D. Lee M. S. Marchetti P. Pietropaolo M. Towns R. Vaccaro M. I. (2011). The emerging role of autophagy in the pathophysiology of diabetes mellitus. Autophagy 7 (1), 2–11. 10.4161/auto.7.1.13044 20935516 PMC3359481

[B16] Group S. S. (2004). SEARCH for diabetes in Youth: A multicenter study of the prevalence, incidence and classification of diabetes mellitus in youth. Control Clin. Trials 25 (5), 458–471. 10.1016/j.cct.2004.08.002 15465616

[B17] Hayat S. Cheng Z. Ahmad H. Ali M. Chen X. Wang M. (2016). Garlic, from remedy to stimulant: Evaluation of antifungal potential reveals diversity in phytoalexin allicin content among garlic cultivars; allicin containing aqueous garlic extracts trigger antioxidants in cucumber. Front. Plant Sci. 7, 1235. 10.3389/fpls.2016.01235 27610111 PMC4996993

[B18] Hyoty H. (2016). Viruses in type 1 diabetes. Pediatr. Diabetes 17 (22), 56–64. 10.1111/pedi.12370 27411438

[B19] Katsarou A. Gudbjornsdottir S. Rawshani A. Dabelea D. Bonifacio E. Anderson B. J. (2017). Type 1 diabetes mellitus. Nat. Rev. Dis. Prim. 3, 17016. 10.1038/nrdp.2017.16 28358037

[B20] Kim Y. H. Kim S. Kim K. A. Yagita H. Kayagaki N. Kim K. W. (1999). Apoptosis of pancreatic beta-cells detected in accelerated diabetes of NOD mice: No role of fas-fas ligand interaction in autoimmune diabetes. Eur. J. Immunol. 29 (2), 455–465. 10.1002/(SICI)1521-4141(199902)29:02<455::AID-IMMU455>3.0.CO;2-A 10064061

[B21] Krischer J. P. Lynch K. F. Schatz D. A. Ilonen J. Lernmark A. Hagopian W. A. (2015). The 6 year incidence of diabetes-associated autoantibodies in genetically at-risk children: The TEDDY study. Diabetologia 58 (5), 980–987. 10.1007/s00125-015-3514-y 25660258 PMC4393776

[B22] Lai L. Chen J. Wang N. Zhu G. Duan X. Ling F. (2017). MiRNA-30e mediated cardioprotection of ACE2 in rats with Doxorubicin-induced heart failure through inhibiting cardiomyocytes autophagy. Life Sci. 169, 69–75. 10.1016/j.lfs.2016.09.006 27633839

[B23] Lawson L. D. Hunsaker S. M. (2018). Allicin bioavailability and bioequivalence from garlic supplements and garlic foods. Nutrients 10 (7), 812. 10.3390/nu10070812 29937536 PMC6073756

[B24] Lenzen S. (2008). The mechanisms of alloxan- and streptozotocin-induced diabetes. Diabetologia 51 (2), 216–226. 10.1007/s00125-007-0886-7 18087688

[B25] Li C. L. Liu X. H. Qiao Y. Ning L. N. Li W. J. Sun Y. S. (2020). Allicin alleviates inflammation of diabetic macroangiopathy via the Nrf2 and NF-kB pathway. Eur. J. Pharmacol. 876, 173052. 10.1016/j.ejphar.2020.173052 32135124

[B26] Li M. Kim D. H. Tsenovoy P. L. Peterson S. J. Rezzani R. Rodella L. F. (2008). Treatment of obese diabetic mice with a heme oxygenase inducer reduces visceral and subcutaneous adiposity, increases adiponectin levels, and improves insulin sensitivity and glucose tolerance. Diabetes 57 (6), 1526–1535. 10.2337/db07-1764 18375438

[B27] Liu Q. R. Aseer K. R. Yao Q. Zhong X. Ghosh P. O'Connell J. F. (2021). Anti-inflammatory and pro-autophagy effects of the cannabinoid receptor CB2R: Possibility of modulation in type 1 diabetes. Front. Pharmacol. 12, 809965. 10.3389/fphar.2021.809965 35115945 PMC8804091

[B28] Lu X. X. Hu Z. W. (2016). New methods to detect autophagic flux. Yao Xue Xue Bao 51 (1), 45–51. 27405161

[B29] Marwick C. (1993). Desperate use' gene therapy guidelines ready. JAMA 269 (7), 843. 10.1001/jama.1993.03500070019004 8426435

[B30] Mihalache C. C. Simon H. U. (2012). Autophagy regulation in macrophages and neutrophils. Exp. Cell Res. 318 (11), 1187–1192. 10.1016/j.yexcr.2011.12.021 22245582

[B31] Miron T. Rabinkov A. Mirelman D. Wilchek M. Weiner L. (2000). The mode of action of allicin: Its ready permeability through phospholipid membranes may contribute to its biological activity. Biochim. Biophys. Acta 1463 (1), 20–30. 10.1016/s0005-2736(99)00174-1 10631291

[B32] Mizushima N. Yoshimori T. Levine B. (2010). Methods in mammalian autophagy research. Cell 140 (3), 313–326. 10.1016/j.cell.2010.01.028 20144757 PMC2852113

[B33] Nadeem M. S. Kazmi I. Ullah I. Muhammad K. Anwar F. (2021). Allicin, an antioxidant and neuroprotective agent, ameliorates cognitive impairment. Antioxidants (Basel) 11 (1), 87. 10.3390/antiox11010087 35052591 PMC8772758

[B34] Nagata S. (1997). Apoptosis by death factor. Cell 88 (3), 355–365. 10.1016/s0092-8674(00)81874-7 9039262

[B35] Nathan D. M. Group D. E. R. (2014). The diabetes control and complications trial/epidemiology of diabetes interventions and complications study at 30 years: Overview. Diabetes Care 37 (1), 9–16. 10.2337/dc13-2112 24356592 PMC3867999

[B36] Notte A. Leclere L. Michiels C. (2011). Autophagy as a mediator of chemotherapy-induced cell death in cancer. Biochem. Pharmacol. 82 (5), 427–434. 10.1016/j.bcp.2011.06.015 21704023

[B37] Osman M. Adnan A. Salmah Bakar N. Alashkham F. (2012). Allicin has significant effect on autoimmune anti-islet cell antibodies in type 1 diabetic rats. Pol. J. Pathol. 63 (4), 248–254. 10.5114/pjp.2012.32772 23359194

[B38] Pan B. Li J. Parajuli N. Tian Z. Wu P. Lewno M. T. (2020). The calcineurin-TFEB-p62 pathway mediates the activation of cardiac macroautophagy by proteasomal malfunction. Circ. Res. 127 (4), 502–518. 10.1161/CIRCRESAHA.119.316007 32366200 PMC7416491

[B39] Panyod S. Wu W. K. Chen P. C. Chong K. V. Yang Y. T. Chuang H. L. (2022). Atherosclerosis amelioration by allicin in raw garlic through gut microbiota and trimethylamine-N-oxide modulation. NPJ Biofilms Microbiomes 8 (1), 4. 10.1038/s41522-022-00266-3 35087050 PMC8795425

[B40] Toygar I. Tureyen A. Demir D. Cetinkalp S. (2020). Effect of allicin on wound healing: An experimental diabetes model. J. Wound Care 29 (7), 388–392. 10.12968/jowc.2020.29.7.388 32654608

[B41] Tuomilehto J. (2013). The emerging global epidemic of type 1 diabetes. Curr. Diab Rep. 13 (6), 795–804. 10.1007/s11892-013-0433-5 24072479

[B42] Vaquero E. C. Rickmann M. Molero X. (2007). Tocotrienols: Balancing the mitochondrial crosstalk between apoptosis and autophagy. Autophagy 3 (6), 652–654. 10.4161/auto.5088 17932465

[B43] Vavra J. J. Deboer C. Dietz A. Hanka L. J. Sokolski W. T. (1959). Streptozotocin, a new antibacterial antibiotic. Antibiot. Annu. 7, 230–235. 13841501

[B44] Wang Y. Bai C. Guan H. Chen R. Wang X. Wang B. (2015). Subchronic exposure to arsenic induces apoptosis in the hippocampus of the mouse brains through the Bcl-2/Bax pathway. J. Occup. Health 57 (3), 212–221. 10.1539/joh.14-0226-OA 25787108

[B45] Wirawan E. Vanden Berghe T. Lippens S. Agostinis P. Vandenabeele P. (2012). Autophagy: For better or for worse. Cell Res. 22 (1), 43–61. 10.1038/cr.2011.152 21912435 PMC3351915

[B46] Wu Y. T. Tan H. L. Huang Q. Kim Y. S. Pan N. Ong W. Y. (2008). Autophagy plays a protective role during zVAD-induced necrotic cell death. Autophagy 4 (4), 457–466. 10.4161/auto.5662 18253089

[B47] Xiang Q. Cheng Z. Wang J. Feng X. Hua W. Luo R. (2020). Allicin attenuated advanced oxidation protein product-induced oxidative stress and mitochondrial apoptosis in human nucleus pulposus cells. Oxid. Med. Cell Longev. 2020, 6685043. 10.1155/2020/6685043 33381267 PMC7758128

[B48] Xu Y. Fu H. Wang Z. Zhou X. Xu Y. Wang D. (2010). Improved methods of isolation and purification of rat islets and its viability research. Zhongguo Xiu Fu Chong Jian Wai Ke Za Zhi 24 (4), 406–409. 20458999

[B49] Yang Z. Klionsky D. J. (2010). Eaten alive: A history of macroautophagy. Nat. Cell Biol. 12 (9), 814–822. 10.1038/ncb0910-814 20811353 PMC3616322

[B50] Yuan K. Huang C. Fox J. Laturnus D. Carlson E. Zhang B. (2012). Autophagy plays an essential role in the clearance of *Pseudomonas aeruginosa* by alveolar macrophages. J. Cell Sci. 125 (2), 507–515. 10.1242/jcs.094573 22302984 PMC3283879

[B51] Yue Z. Jin S. Yang C. Levine A. J. Heintz N. (2003). Beclin 1, an autophagy gene essential for early embryonic development, is a haploinsufficient tumor suppressor. Proc. Natl. Acad. Sci. U. S. A. 100 (25), 15077–15082. 10.1073/pnas.2436255100 14657337 PMC299911

[B52] Zhai N. Wang H. Chen Y. Li H. Viktor K. Huang K. (2018). Taurine attenuates OTA-promoted PCV2 replication through blocking ROS-dependent autophagy via inhibiting AMPK/mTOR signaling pathway. Chem. Biol. Interact. 296, 220–228. 10.1016/j.cbi.2018.10.005 30332612

[B53] Zheng J. Wang Y. Liu Y. Han S. Zhang Y. Luo Y. (2022). cPKCγ deficiency exacerbates autophagy impairment and hyperphosphorylated tau buildup through the AMPK/mTOR pathway in mice with type 1 diabetes mellitus. Neurosci. Bull. 38 (10), 1153–1169. 10.1007/s12264-022-00863-4 35596894 PMC9554100

[B54] Zheng Q. Su H. Ranek M. J. Wang X. (2011). Autophagy and p62 in cardiac proteinopathy. Circ. Res. 109 (3), 296–308. 10.1161/CIRCRESAHA.111.244707 21659648 PMC3142307

[B55] Zinman B. (2015). The international diabetes federation world diabetes congress 2015. Eur. Endocrinol. 11 (2), 66. 10.17925/EE.2015.11.02.66 29632570 PMC5819067

[B56] Zois C. E. Koukourakis M. I. (2009). Radiation-induced autophagy in normal and cancer cells: Towards novel cytoprotection and radio-sensitization policies?. Autophagy 5 (4), 442–450. 10.4161/auto.5.4.7667 19164950

