# Supplementary figures and images for: The protective roles of allicin on type 1 diabetes mellitus through AMPK/mTOR mediated autophagy pathway
Source: Front Pharmacol. 2023 Feb 3;14:1108730. doi: 10.3389/fphar.2023.1108730 (PMC9937553; doi:10.3389/fphar.2023.1108730)

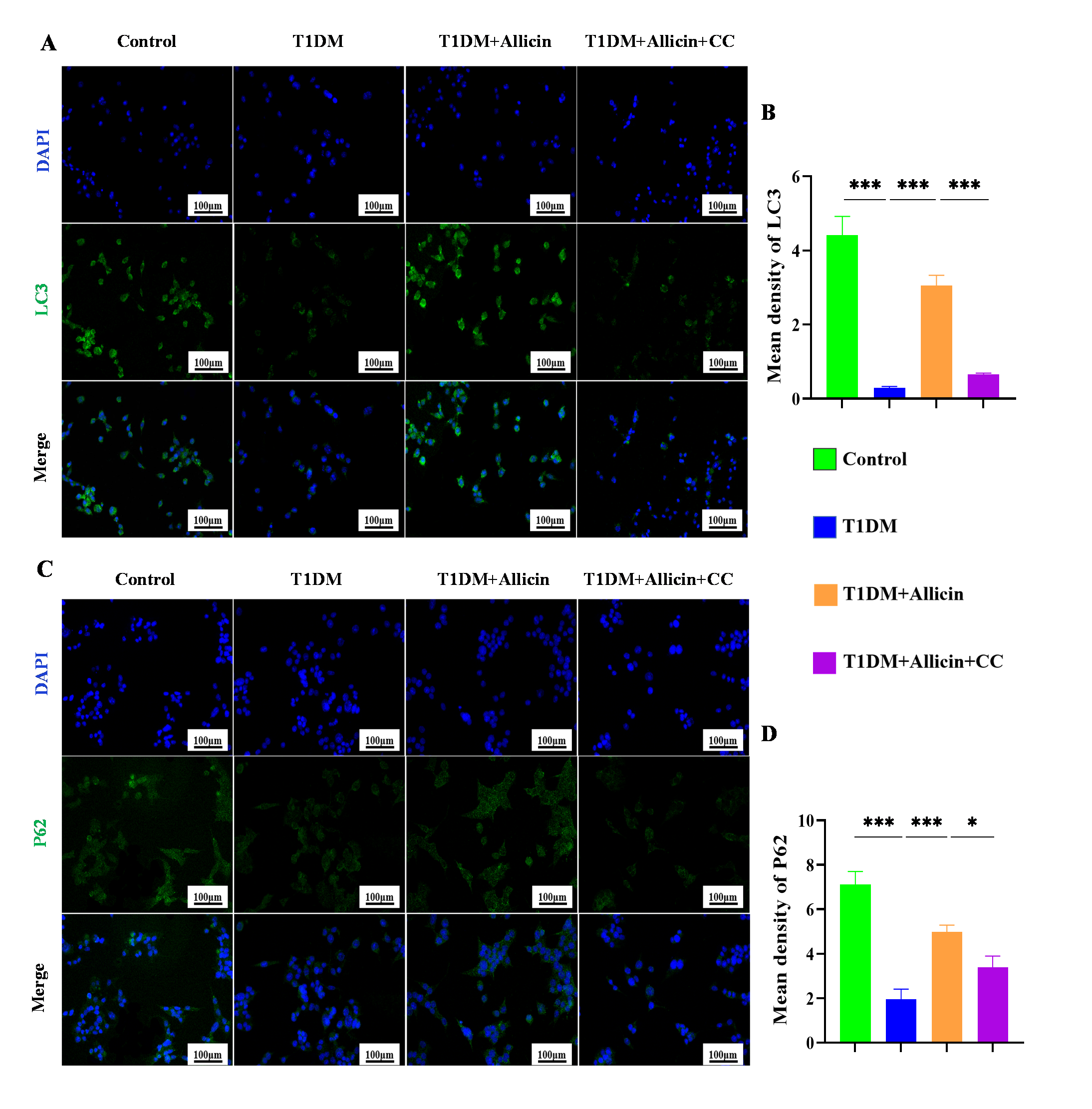

Supplement: Supplementary file 1 [file Image2.TIF]

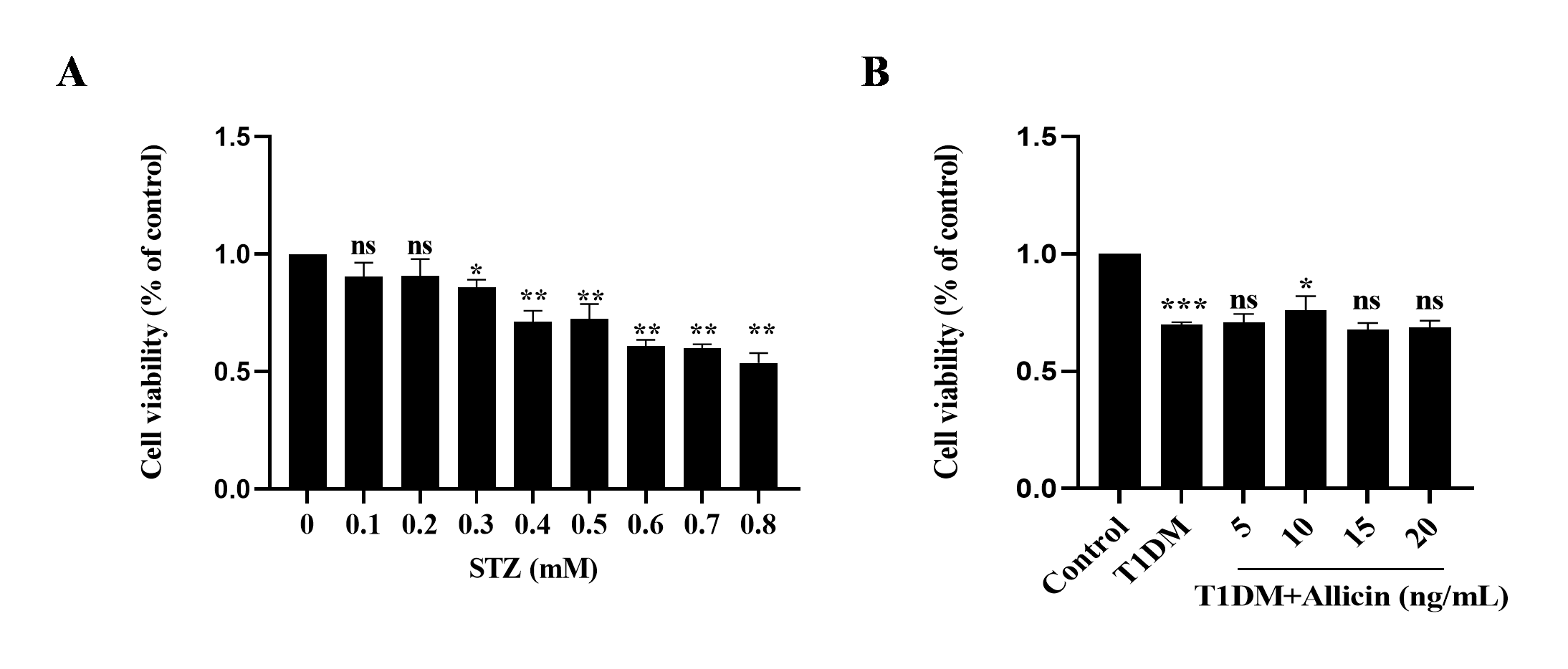

Supplement: Supplementary file 2 [file Image1.TIF]
