# Supplementary material for: The protective roles of allicin on type 1 diabetes mellitus through AMPK/mTOR mediated autophagy pathway
Source: Front Pharmacol. 2023 Feb 3;14:1108730. doi: 10.3389/fphar.2023.1108730 (PMC9937553; doi:10.3389/fphar.2023.1108730)
Supplement: Supplementary file 3 [file Table1.DOC]

**Table 1**. Antibodies

| **Antibody** | **Species** | **Company (catalogue)** | **Dilution** | |
| --- | --- | --- | --- | --- |
|  |  |  | **WB** | **IHC/IF** |
| Insulin | Rabbit | Abcam(ab181547) | 1：1000 | 1: 100 |
| AMPK | Mouse | [Santa Cruz Biotechnology](http://www.baidu.com/link?url=JyKcE01MHDo2C82_vA7PLGsOo6FHhidzFLsl0vS1U__) (sc-398861) | 1: 500 | ND |
| p-AMPK | Rabbit | Cell Signaling Technology (#2535) | 1: 1000 | ND |
| mTOR | Mouse | [Santa Cruz Biotechnology](http://www.baidu.com/link?url=JyKcE01MHDo2C82_vA7PLGsOo6FHhidzFLsl0vS1U__) (sc-517464) | 1: 500 | ND |
| p-mTOR | Mouse | [Santa Cruz Biotechnology](http://www.baidu.com/link?url=JyKcE01MHDo2C82_vA7PLGsOo6FHhidzFLsl0vS1U__) (sc-293133) | 1: 500 | ND |
| BECLIN-1 | Rabbit | Abcam (ab62557) | 1: 5000 | ND |
| LC3 | Rabbit | Abcam (ab192890) | 1: 2000 | 1: 200 |
| P62 | Mouse | [Santa Cruz Biotechnology](http://www.baidu.com/link?url=JyKcE01MHDo2C82_vA7PLGsOo6FHhidzFLsl0vS1U__) (sc-48402) | 1: 500 | 1: 100 |
| BAX | Mouse | [Santa Cruz Biotechnology](http://www.baidu.com/link?url=JyKcE01MHDo2C82_vA7PLGsOo6FHhidzFLsl0vS1U__) (sc-7480) | 1: 500 | 1: 100 |
| BCL-2 | Mouse | [Santa Cruz Biotechnology](http://www.baidu.com/link?url=JyKcE01MHDo2C82_vA7PLGsOo6FHhidzFLsl0vS1U__) (sc-7382) | 1: 500 | 1: 100 |
| β-actin | Rabbit | [Santa Cruz Biotechnology](http://www.baidu.com/link?url=JyKcE01MHDo2C82_vA7PLGsOo6FHhidzFLsl0vS1U__) (sc-47778) | 1:500 | ND |

ND = Not detected; WB = Western blot; IHC: Immunohistochemistry; IF: Immunofluorescence.
