# Supplementary material for: The protective roles of allicin on type 1 diabetes mellitus through AMPK/mTOR mediated autophagy pathway
Source: Front Pharmacol. 2023 Feb 3;14:1108730. doi: 10.3389/fphar.2023.1108730 (PMC9937553; doi:10.3389/fphar.2023.1108730)
Supplement: Supplementary file 4 [file Table2.DOC]

**Table 2. Primer information**

| **Gene name** | **Primer direction** | **Sequences (5’to 3’)** | **PCR**  **(bp)** |
| --- | --- | --- | --- |
| *PDX1* | Forward | AAATCCACCAAAGCTCACGC | 157 |
|  | Reverse | GGGGCCGGGAGATGTATTTG |  |
| *INS1* | Forward | CAAACCCACCCAGGCTTTTG | 183 |
|  | Reverse | AACGCCAAGGTCTGAAGGTC |  |
| *GLUT2* | Forward | CGGGGACAAACTTGGAAGGA | 201 |
|  | Reverse | CTGAGTGTGGTTGGAGCGAT |  |
| *Bax* | Forward | ACACTGGACTTCCTCCGTGA | 70 |
|  | Reverse | AGAGGAGGCCTTCCCAGC |  |
| *Bcl-2* | Forward | TGAACTGGGGGAGGATTGTG | 227 |
|  | Reverse | CAGAGACAGCCAGGAGAAATCA |  |
| *GAPDH* | Forward | GGCAAATTCAACGGCACAGT | 195 |
|  | Reverse | CCTTTTGGCTCCACCCTTCA |  |
